# Effects of Various Drying Methods on Some Physico-Chemical Properties and the Antioxidant Profile and ACE Inhibition Activity of Oyster Mushrooms (*Pleurotus Ostreatus*)

**DOI:** 10.3390/foods9020160

**Published:** 2020-02-07

**Authors:** Sergey Piskov, Lyudmila Timchenko, Wolf-Dieter Grimm, Igor Rzhepakovsky, Svetlana Avanesyan, Marina Sizonenko, Vladimir Kurchenko

**Affiliations:** 1 Institute of Live Science, North Caucasus Federal University, 355017 Stavropol, Russia; piskovsi77@mail.ru (S.P.); l_timchenko@mail.ru (L.T.); 78igorr@mail.ru (I.R.); s.avanesan@yandex.ru (S.A.); risha_veresk@mail.ru (M.S.); 2 Periodontology, School of Dentistry, Faculty of Health, Witten/Herdecke University, A.-Herrhausen-Street 50, 58448 Witten, Germany; 3 Faculty of Biology, Belarusian State University, 220030 Minsk, Belarus; kurchenko@tut.by

**Keywords:** drying, oyster mushrooms, antioxidant activity, ACE inhibitory activity

## Abstract

In food biotechnology, *Pleurotus ostreatus* is of great interest as a source of natural antioxidants and angiotensin-converting enzyme (ACE) inhibitors. However, research in this area has not yet been completed. The effect of various drying methods on the structural properties and the rehydration capacity of mushrooms was investigated in this paper. The content of secondary metabolites, the peptide profile, and the antioxidative effect and ACE inhibitory activity of dry mushrooms were investigated in vitro, simulating the process of gastrointestinal digestion. X-ray microtomography has confirmed that structure of lyophilic and sun-dried mushrooms is dominated by open pores, and in mushrooms dried with hot air and microwave, closed pores. Experiments have shown that the conditions of freeze drying and sun drying of *Pleurotus ostreatus* provide a higher rehydration capacity of dried mushrooms. The maximum activity of radical absorption of the oyster mushroom after microwave drying was observed. The iron restoring capacity of the mushrooms is maximally maintained with microwave drying and hot-air drying. The properties of the antioxidant product with an emphasis on the high activity of inhibiting lipid oxidation of the mushroom maximized after drying in the sun. Mushrooms dried lyophilically and in the sun showed the highest ACE inhibitory activity.

## 1. Introduction

Recently, interest in natural antioxidants and their use in the production of a new generation of foods has increased. Epidemiological studies [1] have highlighted the close link between foods that are rich in antioxidants Reactive oxygen species (ROS) with systemic health diseases [2]. ROS, especially when human in vivo antioxidant defense and immunological repair systems are inadequate to prevent the damage. Oxidative stress has been associated with hypertension development [3]. If hypertension persists over a long period of time, this is one of the risks for strokes, heart disease, and eventually for the emergence of chronic kidney failure. 

According to recent studies, oxidative stress is involved in the mechanisms of metabolic syndrome [4]. Metabolic syndrome, in turn, can be a leading risk factor for cardiovascular disease, type II diabetes mellitus, reproductive pathology and several common cancers [5]. A parallel study provided by our international research team on the combination of metabolic syndrome (MS) and chronic obstructive pulmonary disease (COPD) revealed pathological mechanisms linking MS and COPD [6]. 

The intake of exogenous antioxidants is critical to maintaining adequate levels of antioxidants to ensure balance with prevention of the aforementioned pathologies.

Special attention is paid in this context to the mushrooms. Due to the large number of biologically active substances they are of interest as valuable raw materials for functional products, especially those with antioxidant effects [7,8]. Oyster mushrooms are promising in this respect as their fruiting bodies are readily available and rich in natural antioxidants [9,10]. 

However, it should be noted that the content and efficacy of biologically active compounds in mushrooms vary widely, not only depending on the species, growing conditions and maturity [11,12], but also on storage and processing methods [13,14].

The fruiting bodies of *Pleurotus ostreatus* are transient. Due to the high-water content (87–95%), oyster mushrooms, like other mushrooms, cannot be stored for more than 24 h in the environment and 5–7 days after cooling, which requires rapid treatment [15].

Drying remains the most common method for the long-term preservation of mushrooms. Dehydration prevents the growth of microorganisms, reduces enzyme activity, slows down reactions in the presence of water and thus effectively preserves the positive properties of mushrooms and prolongs their shelf life [16]. However, due to various dehydration conditions and mechanisms, and the different composition and types of raw materials, the drying process may have a mixed influence on product properties [17]. Drying processes can influence not only the original properties and composition, but also the cell structure of the raw material and thus the release and bioavailability of substances from the food matrix [18,19].

Certain drying methods can improve the quality, preservation, and even value of raw materials [20,21]. Others, however, may be accompanied by a significant decline [22,23]. Therefore, the technology for obtaining dry natural products must be developed separately for each type of raw material and based on experimental data on the influence of specific drying conditions on the specific properties of the final product. This is particularly important for mushroom raw materials with low resistance to mechanical and physical factors compared to plant material [24].

The most common applications in the food industry are lyophilic drying, hot air drying, microwave drying and sun drying. Each of the above drying methods has its own unique characteristics, but the comparative analysis of these methods is not sufficiently studied regarding their impact on the structural, physical, chemical and functional properties of *Pleurotus ostreatus* mushrooms. In particular, comparative publications on the influence of the mentioned drying methods on the antioxidant activity of fruit bodies of *Pleurotus ostreatus* with an emphasis on specific links in the process of free-radical oxidation, as well as on ACE-inhibiting properties are lacking.

Therefore, the aim of this study was to study and compare the effects of four different drying methods (lyophilic, hot air, microwave, and solar drying) on the structural properties, rehydration capacity, chemical composition with an emphasis on phenols, flavonoids, profile of secondary metabolites and peptides, antioxidant and ACE-inhibiting activity of *Pleurotus ostreatus*. For the first time, the use of different drying methods was aimed at selecting dehydration conditions that provide maximum antioxidant properties (antiradical scavenging activity, reducing power activity, ferric reducing ability, total antioxidant capacity, activity of inhibiting lipid oxidation) and ACE-inhibiting effect of *Pleurotus ostreatus* under conditions of experimental gastrointestinal digestion.

## 2. Materials and Methods 

### 2.1. Raw Materials

The subject of this study was a common oyster mushroom (*Pleurotus ostreatus*), strain NK35 (SYLVAN, Hungary, Dunaharaszti) from the 2019 harvest, cultivated under mushroom growing conditions in the Stavropol region. For the study, fruit bodies of the same size and maturity without mechanical damage were selected. The initial moisture content of the fresh oyster mushrooms selected for the study was 88.56 ± 0.3%.

### 2.2. Chemicals

Chemicals were obtained from the following sources: Pepsin from porcine gastric mucosa (activity 600−1800 U/mg), hydrochloric acid 37%, sodium hydroxide ≥98% (Sigma-Aldrich, St. Louis, MI, USA), pancreatin (activity): Amylase 22,500 FIP E/g, lipase 22,500 FIP E/g, protease 1050 FIP E/g, AppliChem, Darmstadt, Germany), bile extract (millipore), petroleum ether 40–60 °C, ≥90%, formaldehyde, sulfanilic acid 99%, peptone, picric acid ≥98.0%, sodium carbonate 99.5% (Sigma-Aldrich), d-(+)-glucose monohydrate (AppliChem); Folin-Denis reagent, gallic acid (Supelco); ethyl alcohol 96.0–97.2%, aluminium chloride 99.9%, sodium acetate >99%, quercetin, phosphate buffered saline solution (pH 7.2 (25 °C), potassium ferrocyanide ≥98.8%, trichloroacetic acid ≥99%, iron chloride ≥99%, ascorbic acid ≥99%, 1,10-phenanthroline ≥99%, ammonium ferric sulphate dodecahydrate 99% (Sigma-Aldrich); antioxidant test kit (Institute of Bioorganic Chemistry, National Academy of Sciences of Belarus, Republic of Belarus); metmyoglobin, ABTS (2,2′-Azino-bis(3-ethylbenzothiazolin-6-sulphonic acid) diammonium salt), hydrogen peroxide solution 30 wt.-(*w*/*w*)%. in H_2_O, Trolox (6-hydroxy-2,5,7,8-tetramethylchroman-2-carboxylic acid 97%, oleic acid ≥99%, ammonium thiocyanate ≥97.5% (Sigma-Aldrich); *N*-Hippuryl-His-Leu hydrate-powder ≥98%; ACE from rabbit lung ≥1.0 units/mg; ethyl acetate 99.8%; hippuric acid 98%, formic acid ~98%, acetonitrile isocratic grade for liquid chromatography, α-Cyano-4-hydroxycinnamic acid, trifluoroacetic acid >99% (Sigma-Aldrich); standards for chemical research were obtained from Diaem (Moscow, Russia).

### 2.3. Drying Process

Before drying, the mushrooms were washed under running water at room temperature for 30 s. The remaining water was removed with a paper towel. The entire mushroom was dried in four various ways: freeze drying (FD), hot air drying (HAD), microwave drying (MWD), and sun drying (SD).

In each experiment, 200 g of oyster mushrooms were dried. The drying process was carried out until a constant moisture content was achieved in the samples. The initial and final moisture content of the samples was measured with a moisture analyzer (MB 25, Ohaus Corporation, Parsippany, NJ, USA) with an accuracy of 0.001%. The values were considered when selecting the automatic measuring option with a heating temperature of 100 °C and a measuring time of 5 min.

The final moisture content of the mushrooms FD, HAD, MWD and SD was 3.85 ± 0.1%, 7.75 ± 0.1%, 5.26 ± 0.1%, 7.33 ± 0.1%.

The finished dry oyster samples were stored in hermetically sealed containers and stored in a dark place at a temperature no higher than 25 °C for further analysis.

#### 2.3.1. Freeze Drying (FD)

The first part of the mushrooms was frozen in the freezer (TEFCOLD SE-45, Viborg, Denmark) at −40 °C for 72 h. It was then dried in a LS-500 freeze dryer (Prointech, St Petersburg, Russia) with a sublimator and a vacuum station. The transparent lid of the drying chamber was covered with aluminium foil to prevent the degradation of antioxidants by light oxidation.

The average operating pressure in the drying chamber reached 80.0–90.0 Pa, the condenser temperature −49.0–50.0 °C, the mushroom heating for the entire drying process did not exceed 30 °C. The average drying time was 26–27 h.

#### 2.3.2. Hot Air Drying (HAD)

The second part of the mushrooms was dried at atmospheric pressure and temperature by 55 °C in a drying cabinet model FD 115 (Binder, Germany) with forced ventilation. The mushrooms in the dryer were placed in a layer on trays. Temperature and air velocity were kept constant at 50 °C and 1.3 m/s, respectively.

#### 2.3.3. Microwave Drying (MWD)

One third of the mushrooms were dried in the microwave in the household (model WD 900 EL 23-2III, Erisson, Russia). The single-layer sample arrangement on the turntable ensured uniform heating. Microwave drying was performed at a power of 200 W for 40 min.

#### 2.3.4. Sun Drying (SD)

The fourth batch of mushrooms was placed in stainless steel dishes and dried in the open sun at an ambient temperature of 25 ± 5 °C and a relative humidity of 40 ± 5% for 3 days (average 9 h per day).

### 2.4. Determination of Structure and Rehydration Ratio 

#### 2.4.1. X-ray CT

Volumetric structural properties of dry fruit bodies of the *Pleurotus ostreatus* were investigated by X-ray microtomography with an X-ray microcomputer tomography system Skyscan 1176 (Bruker, Kontich, Belgium). The study selected cylindrical fragments of *Pleurotus ostreatus* with a diameter of 6–7 mm and a length of 9–10 mm, which were cut from the stem of the mushroom directly under the cap. The samples were scanned with the following parameters: X-ray voltage and current of 40 ΚV and 600 μA respectively; without filter; pixel size of 8.77 µm; scanning time of 60 min. The scan protocol included a 180° rotation at 0.3° rotation. The exposure time is 535 ms per image, the image averaging is 4.

The three-dimensional reconstruction of the samples was performed with the reconstruction software NRecon (version 1.7.1.0, Bruker, Kontich, Belgium). The following settings were used for the microtomographic reconstruction: without smoothing, correction of ring artefacts = 10% and correction of beam hardening = 51%.

CTAn software was used for the quantitative analysis of the internal structure (version: 1.18.4.0, Bruker Kontich, Belgium). The internal structure of *Pleurotus ostreatus* samples of dried fruit bodies was characterized by the following microtomographic parameters used for food [25]: percent object volume (POV); objective surface-to-volume ratio (OSVR); fragmentation index (FI); degree of anisotropy (DA); structure modelling index (SMI).

#### 2.4.2. Determination of Rehydration Ratio (RR)

RR of dried mushrooms was determined by immersing pre-weighed samples in distilled water (50 mL water per 1 g dry mushrooms) at room temperature. The mushrooms were then removed from the water and gently soaked with a paper towel to remove excess water from the surface. Mushroom samples were weighed on a ML203E precision scale (Mettler Toledo, Spain). The evaluation was carried out every 5 min until reaching the mass transfer equilibrium. Each rehydration experiment was repeated three times. RR were calculated using the formula [17]:

RR = (W_1_ − W_0_)/W_0_, (1)

where W_1_ and W_0_ are masses of rehydrated or dried oyster mushroom samples.

### 2.5. In Vitro Digestion Methods

Chemical characterization and study of the antioxidant properties of fruiting bodies of *Pleurotus ostreatus*, dried in various ways, were performed in vitro by simulating the process of gastrointestinal digestion, which was reproducible by the method of McDougall et al. [26] in some of its changes.

At 5 g lightly ground dried fruit bodies, 100 mL distilled water was added to the oyster mushrooms, then 33% HCl solution up to 0.5% was added and the mixture incubated with 0.1% pepsin in ES 20/60 (Biosan, Latvia) thermal shaker for 120 min at 37 °C at shaking temperature in 120 rpm mode. Neutralized 1 Μ NaOH to pH 7.0 (pH meter S400-B, Mettler Toledo, Spain), addition of a mixture of pancreatin 2 mg/mL and 4 mL bile extract, incubation in a thermoshaker for 120 min at 37 °C and shaking 120 rpm. The extraction was stopped by boiling for 10 min.

The extracts were centrifuged for 60 min at 8000 rpm (centrifugal table Universal 320, Hettich, Germany). 

After centrifugation, the sludge was separated, and petroleum ether was added to the supernatant (1:3). Mixed with a magnetic stirrer for 4 h. The water and lipid fractions were separated in the separating funnel. All subsequent analyses were performed with the water fraction of the extract.

### 2.6. Chemical Analysis of Oyster Mushroom Extracts

#### 2.6.1. Determination of the Basic Chemical Parameters 

The degree of hydrolysis (DH%) was determined by the AN/TN ratio in the final substance, where AN is the amount of amine nitrogen determined by the formaldehyde titration method [27], TN is the amount of total nitrogen determined by the Kjeldahl titrimetric method.

The dry mass was determined with the moisture analyzer Ohaus MB 25 (Ohaus Corporation, USA) in automatic mode at 100 °C. Ionometry was performed with the pH meter S400-B (Mettler Toledo, Spain).

Peptide concentrations were determined by the biuret method [28] using 1% peptone solution as standard. The optical density of extract samples was measured at a wavelength of 540 nm with the UV spectrophotometer SF 102 (NPO INTERFOTOFIZIKA, Moscow, Russia). 

The amount of sugar in extracts of dried mushrooms was spectrophotometrically determined by reaction with picric acid (Creselius-Seifert). In the interaction of reducing sugars with picric acid, they are oxidized to the corresponding acids, and the picric acid is returned to the red or brownish-red picric acid. For this purpose, 1 mL of saturated aqueous picric acid solution and 3 mL of sodium carbonate solution were added to 0.5 mL of the tested mushroom extracts, then the tube was transferred to a boiling water bath for 30 min. Cool to room temperature and bring to 10 mL with distilled water. The optical density of the solution was determined at 455 nm (blue filter). The reducing sugar content was calculated from the calibration curve consisting of standard glucose solutions.

#### 2.6.2. Total Phenol Content (TPC) 

The total phenol content was measured with the Swain, & Hillis method [29] with some modifications. Samples of the extracts obtained in a volume of 2.5 mL were mixed with 0.5 mL Folin Denis Reagent (F.D.R.). After 3 min 0.5 mL of 20% sodium carbonate solution were added. The total volume was increased to 10mL with distilled water. The mixture was incubated in the dark for 90 min. The optical density was then measured at 725 nm using the SF-102 spectrophotometer. Gauliensic acid was used to obtain a standard curve with a calibration range of 0–100 μg/mL (R^2^ = 0.998) and the results were given in mg of the equivalent of Gauliensic acid (GAE) per 1 g mushroom per dry weight (mg GAE/g).

#### 2.6.3. Total Flavonoid Content (TFC)

The content of flavonoids was determined using the colorimetric method described by Chia-Chi et al. [30] in some modifications. 0,5 mL mushroom extract were selected and mixed with 1.5 mL 95% ethanol, 0.1 mL 10% aluminium chloride, 0.1 mL 1M sodium acetate and 2.8 mL distilled water. This mixture was incubated at room temperature for 30 min in the dark. The optical density was measured at 415 nm. Quercetin was used to obtain a standard curve with a calibration range of 0–100 μg/mL and the flavonoid content was calculated in mg quercetin equivalent per gram of dried sample (mg QE/g).

#### 2.6.4. Gas Chromatographic Analysis

The composition of the secondary metabolites was investigated using a gas chromatograph Agilent 6850 (Agilent Technologies, Moscow, Russia) with a mass detector Agilent 5975V. The percentage composition of the secondary metabolites was calculated using peak ranges without the use of correction factors. Qualitative analysis was based on the comparison of mass spectra of substances with the corresponding data of the NIST0.5a mass spectra library.

#### 2.6.5. Matrix-Assisted Laser Desorption/Ionization (MALDI) Time-of-Flight (TOF) Mass Spectrometry

Analysis of the peptide profile of the extracts was performed using MALDI-TOF mass spectrometry. The extracts were centrifuged at 10,000 rpm for 4 min (MiniSpin microcentrifuge (Eppendorf AG, Hamburg, Germany). The supernatant (1 microliters) was deposited on the plate. Pretreated and untreated samples were overlaid with 1 microliters of matrix solution (saturated solution of a-cyano-4-hydroxycinnamic acid in 50% acetonitrile and 2.5% trifluoroacetic acid). The matrix sample was cocrystallized by air drying at room temperature. Measurements were performed with a Microflex mass spectrometer (Bruker Daltonik, Bremen, Germany) using Daltonics FlexControl software (version 3.3.64, Bruker Daltonik, Bremen, Germany). Spectra were recorded in the positive linear mode (laser frequency, 60 Hz; ion source 1 voltage, 19.4 kV; ion source 2 voltage, 17.3 kV; lens voltage, 8.0 kV; mass range, 0 to 20,000 dalton (Da)). For each spectrum 4000 shots from different positions of the target spot (automatic mode) were collected and analyzed. Protein identification was performed using the BIOPEP-UWM database [31].

### 2.7. Determination of Antioxidative Activity 

#### 2.7.1. ABTS Radical Scavenger Activity

The ABTS radical scavenger activity was determined with a set of reagents to quantify the antioxidant activity of FITCHEM (Belarus) with some modifications. A chromogen was prepared by mixing the solutions (metmyoglobin (5 mL, 2.8 μM) dissolved in 5 mL phosphate buffer (50 mM, pH 7.4) and ABTS (2,2-azino-bis-[3-ethylbenzthiazolin-6-sulfonic acid]-diammonium salt, 5 mL, 546 μM) dissolved in 5 mL phosphate buffer).Add 0.9 mL chromogen, 0,3mL substrate (hydrogen peroxide (0.88 mM) to 0,45 mL phosphate buffer (50 mM, pH 7,4) and incubate for 3 min. 0.02 mL sample was added to the resulting solution and kept for 10 min. Absorption was measured at 735 nm. A solution of Trolox (6-hydroxy-2,5,7,8-tetramethylchroman-2-carboxylic acid) was used as standard. In the negative control, distilled water was used instead of extract. The ABTS method was determined with a UV spectrophotometer SF 102.

#### 2.7.2. Reduction of Performance Activity

The analysis was performed using the method described by Klompong et al. [32], based on the principle of increasing the absorption of reaction mixtures. The extract in the volume of 0.2 mL of each sample was mixed with 2 mL phosphate buffer (0.2 M, pH 6.6) and 5 mL 1% potassium hexacyanoferrate. After incubation at 50 °C, 1mL of 10% trichloroacetic acid was added for 20 min and after shaking, the solution was centrifuged for 10 min with a centrifuge (MicroCL 17R, Thermo, Germany) at 2000× *g*. The solution was then mixed with 1 mL of trichloroacetic acid for 20 min. The supernatant was mixed with 2 mL distilled water and 0.5 mL 0.1% ferric chloride. After incubation at room temperature, the optical density at 700 nm was measured with an SF-102 UV spectrophotometer for 10 min. The higher absorption indicates a higher regenerability. Ascorbic acid solution (1 mg/mL) was a positive control.

#### 2.7.3. Ferri Reducibility (the FRAP Assay)

For FRAP analysis, a complex reagent was prepared by dissolving 0.1980 g 1,10-phenantroline in 30–40 mL distilled water at low heat and 0.2892 g iron-ammonium aluminium with 2 mL hydrochloric acid (1 mol/dm^3^) in 30−40 mL distilled water at low heat for 0.1980 g 1,10-phenantroline. The solutions obtained were mixed and the volume increased to 100 mL with distilled water. The prepared reagent was kept for 12 h. Add 2 mL of the complex reagent by 0.02 mL of the extract sample and increase the total volume to 50 mL with distilled water. The mixture was stirred and after 60 min the optical density was measured at a wavelength of 490 nm in cells with an optical path length of 10 mm relative to the distilled water. Ascorbic acid solution (1 mg/mL) was a positive control.

#### 2.7.4. Determination of Total Antioxidant Capacity by Electrochemical Method

The total antioxidant capacity was determined by the electrochemical method to correspond to Piljac-Žegarac et al. [33], and Hoyos-Arbelaez et al. [34]. The total antioxidative capacity was determined using the “Tsvet-Yauza-01-AA” liquid chromatograph with amperometric detection (Russia). Gallic acid solution was used as reference substance. Amperometrically, the method is based on measuring the strength of the electric current generated by oxidation of the antioxidant molecules on the working electrode surface at a certain potential, which is converted into a digital signal after amplification. During the analysis, a calibration curve for 5 consecutive measurements of 5 calibration solutions of Gallic acid is prepared. The result is the arithmetic mean of 5 measurements. Based on the obtained data, a calibration diagram is created, which is described by the equation:

Y = aX + b,(2)

The calibration diagram is then calculated from the calibration solutions. In the coordinates: X is the mass concentration of gallic acid, mg/L; Y is the signal of gallic acid (peak area), nA/s.

#### 2.7.5. Lipid Peroxidation Inhibition Test

The activity of lipid peroxidation inhibition was measured in the oleic acid emulsion system using the method described by Kimatu et al. [35] in some changes. To 4.0 cm^3^ phosphate buffer (50 mm, pH 7.0) 0.1 cm^3^ extract and 0.1 cm^3^ oleic acid were added to 4.0 cm^3^ ethanol (95 wt.% water solution). The total volume was brought to 10.0 cm^3^ by distilled water, mixed in a hermetically sealed conical tube with screw cap and incubated for 7 days at 40 °C in the dark. The degree of oxidation was estimated by iron thiocyanate at 24-h intervals. The reaction solution (100 µL) was mixed with 4.7 cm^3^ ethanol (75 wt.%, aqueous solution), 0.1 cm^3^ ammonium thiocyanate (30% wt.%) and 0.1cm^3^ ferric chloride (II) (2mM in 3.5% (vol./r) HCl). After 3 min the absorption capacity at a wavelength of 500 nm was measured with a UV spectrophotometer. The increase of the optical density meant an increase of the degree of oxidation of the oleic acid. Trolox (0.95 mmol/dm^3^) was used as a reference for the investigated activity. In the blank, deionized water was used instead of extract.

### 2.8. Angiotensin-Converting Enzyme Assay (ACE) Inhibitory Assay

The ACE inhibitory activity assay was performed according to the method by Abdullah et al. [36] with some modifications. Briefly, 200 μL of 5mM hippuryl-l-histidine-l-leucine (HHL) (Sigma) solution was mixed with 50 μL of each mushroom extract and the mixture was preincubated at 37 °C for 3 min. The reaction was initiated by the addition of 20 μL of 0.1 U/mL ACE (Sigma) solution and the mixture was again incubated at 37 °C for 30 min. The reaction was terminated with the addition of 250 μL of 1 N HCl. Then, 1.5 mL of ethyl acetate was added to extract the hippuric acid liberated by the reaction. The solution was centrifuged for 10 min; the ethyl acetate layer was then aspirated and evaporated under vacuum condition. The dried hippuric acid was re-dissolved in 1 mL of distilled water and measured spectrophotometrically at 228 nm. The ACE inhibitory activity of the mushroom extracts was determined by the following equation:

(3) $$\mathrm{Percentage}\;\mathrm{of}\;\mathrm{inhibition}\;(\%)\;=\;\frac{B-A}{B-C}\times 100,$$

where A is the absorbance of ACE and mushroom extracts, B is the absorbance of ACE and HHL, and C is the absorbance of HHL only.

### 2.9. Statistical Analysis

All statistical analyses were performed with GraphPad Prism for Windows, Version 6.01 (GraphPad Software, San Diego, CA, USA). The statistical analysis was performed by a one-way variance analysis (ANOVA). *p* < 0.05 was considered statistically significant. The results were expressed as mean ± SD (*n* = 3).

## 3. Results and Discussion

**Figure 1:**
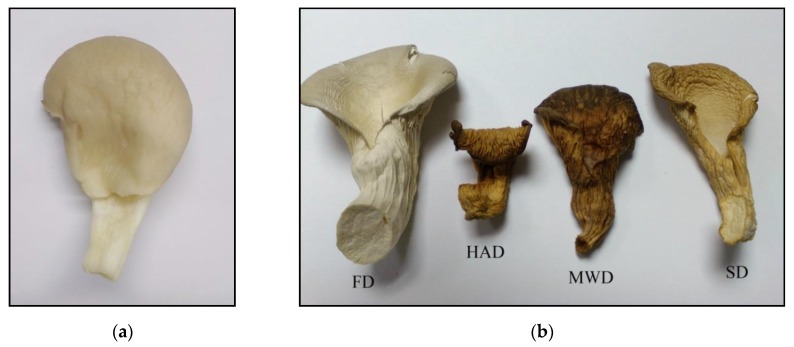
Macroscopic photos of fresh oyster mushroom (**a**) and after drying (**b**) FD—freeze drying; HAD—hot air drying; MWD—microwave drying; SD—sun drying.

It is well known that chromaticity is one of the criteria for the quality of a dry product, the modification of which is due to pigment degradation and enzymatic darkening during drying [37]. Visually lyophilic dried oyster mushrooms have the same colour as fresh mushrooms (Figure 1). 

Dried mushrooms were distributed in the following order of color intensity in the order of their increase: FD < SD < HAD < MWD.

The low colour intensity of FD samples can be explained by the low oxygen concentration in the vacuum range of the lyophilic dryer and thus by the lower intensity of the enzymatic reactions, which are the main cause of the discoloration of dried mushrooms [16]. Darker staining of MWD and HAD samples may be due to the reaction of saccharoamine condensation (Maillard reaction) during heating, as mentioned by Izli & Isik [38].

### 3.1. X-ray CT Analysis

It is known that drainage conditions influence the structure of the final product. Using X-ray microtomography it was reported that freeze-dried mushrooms had uniform pore volume distribution. Dry-air dehydration method lead to the formation of larger cavities [39]. Structural changes can influence the process of mass and heat transfer, accompanied by biochemical changes, as well as the degree of release of bioactive substances from the food matrix [40]. In this context, in order to fully understand the influence of the conditions of the investigated drying methods on the antioxidative properties of *Pleurotus ostreatus*, the structure of dry mushrooms was investigated by X-ray microtomography.

**Figure 2:**
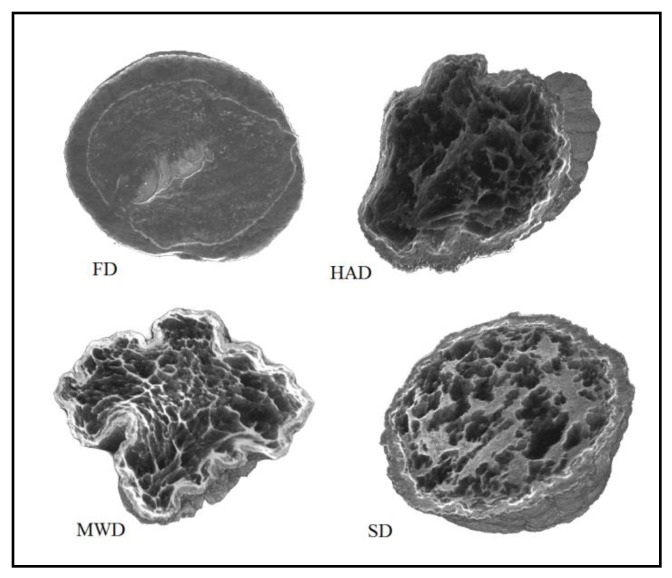
X-ray microtomography images of mushroom samples after drying. FD—freeze drying; HAD—hot air drying; MWD—microwave drying; SD—sun drying.

Figure 2 shows microtomographic images of dry oyster mushroom cross sections. The obtained 3D models of mushroom samples show the spatial distribution of X-rays, with brighter areas corresponding to a higher density and darker areas to cavities.

The microtomographic images show that all samples of dry mushrooms are heterogeneous in structure and number of pores. There is an increase in voids in the direction of FD < SD < MWD < HAD.

Table 1 shows average values and results of statistical analysis of microstructural parameters of dry coat of arms samples obtained with the CTAn software (Skyscan, Kontich, Belgium).

The POV is calculated as the average percentage of pores inside the object. According to the table, the FD samples have the maximum POV value (highest porosity). MWD and SD samples have the lowest POV values.

With respect to the FI index, the structural cohesion index and the measure of relative convexity or concavity of the total pore surface, the FD and SD samples were characterized by negative values of this parameter. This indicates that a large part of their structure is open, cross-linked pores. The HAD and MWD samples had positive FI values, confirming the presence of a high percentage of closed, clearly separated pores in their structure.

The degree of anisotropy (DA) is an indicator of three-dimensional structural symmetry, which in this case indicates the presence or absence of a preferred pore orientation of the samples in several direction [41]. A value of 0 corresponds to full isotropy, a value of 1 corresponds to total anisotropy. According to the mentioned parameters, MWD samples are significantly differentiated, which are characterized by maximum values of DA and SMI parameters. The above mentioned changes quantitatively confirm the higher deformation of the MS mushroom compared to other samples and are consistent with the materials of Apati et al. [42] which states that high drying temperatures lead to an increase in the water release rate and thus to a significant deformation of the structure of mushrooms.

Thus, various drying mechanisms of *Pleurotus ostreatus* fruit bodies have a very different influence on the microstructural properties of the dry product. X-ray microtomography and the obtained data of the three-dimensional microstructure of dried mushrooms proved to be useful tools. Such a plan to identify the internal structure is important as changes in microstructural relationships can influence the physicochemical, nutritional, and functional properties of the product from a range of data [19].

### 3.2. Effects of Drying Methods on the Rehydration Ratio

**Figure 3:**
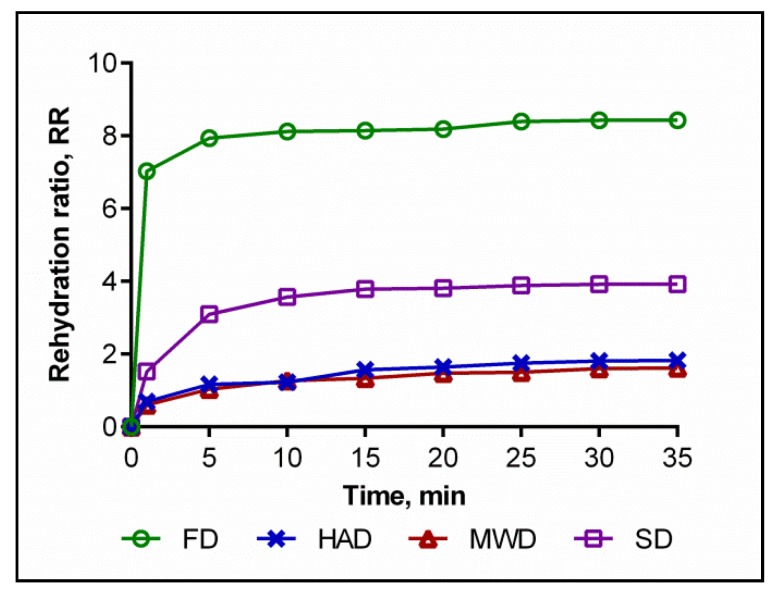
Rehydration curves of dried oyster mushrooms at distinct time. FD—freeze drying; HAD—hot air drying; MWD—microwave drying; SD—sun drying.

Microtomographically recorded structural differences between samples of dry fruit bodies of *Pleurotus ostreatus* were combined with different manifest rehydration properties, which represent a kind of measure of structural damage to the product in the dehydration process (Figure 3).

The RR values of FD mushrooms were found to be maximum and significantly higher than those of other samples. Similar results were found in previous studies [39] and are explained by the above structural differences in dry oyster mushroom samples.

The RR samples of HAD and MWD were relatively low and did not differ significantly. The ability to rehydrate SD samples was mediocre. Similar data are available in the countries of Singh and Pandey [43] or Seremet et al. [44] and may be caused by the structural collapse of tissues and the formation of hard layers in the process of product dehydration to prevent rehydration.

Thus, the conditions of FD and SD may provide a higher rehydration capacity of the dry oyster mushroom product compared to HAD and MWD and thus contribute to shortening the cooking time of mushrooms, facilitating their incorporation and improving the efficiency of the extraction of biologically active substances.

### 3.3. Influence of Drying Processes on Some Chemical Properties of Pleurotus Ostreatus under In Vitro Conditions Simulating the Gastrointestinal Digestive Process 

#### 3.3.1. Basic Chemical Parameters

The net efficacy of food-borne bioactive substances depends on their actual content and activity in the digestive tract [45]. Therefore, a full assessment of the biologically active properties of foods or their ingredients should be carried out under digestive conditions. 

The dry mushroom samples we examined were exposed to reactions that mimic the gastrointestinal digestive process. The basic physicochemical parameters characterizing the extracts obtained under these conditions are listed in Table 2. 

According to the tabular data for amino acids the extract samples were distributed as follows in order to increase their quantity: MWD < FD < HAD < SD. The maximum degree of hydrolysis was characterized by HAD mushrooms, the minimum, MWD. The number of peptides was leading in MWD extracts. The order of decrease of peptide concentration in the rest of the samples looked like this: FD ˃ SD ˃ HAD.

On the one hand, these values show that the conditions of HAD and SD, characterized by a prolonged process of dehydrogenation and possible reactions of autohydrolysis of proteins in the department with the subsequent conditions of simulated process of gastrointestinal digestion, offer a more complete proteolysis of proteins of mushroom tissue. On the other hand, there is evidence of the accumulation of peptides in FD and MWD samples which, according to Sun et al. [46], may provide a high antioxidant effect of *Pleurotus ostreatus* dried fruit extracts.

#### 3.3.2. Effects of Drying Processes on the TPC and TFC of Oyster Mushroom Extracts 

Phenolic compounds are one of the most important groups of secondary metabolites of mushrooms with proven antioxidant properties [47].

**Figure 4:**
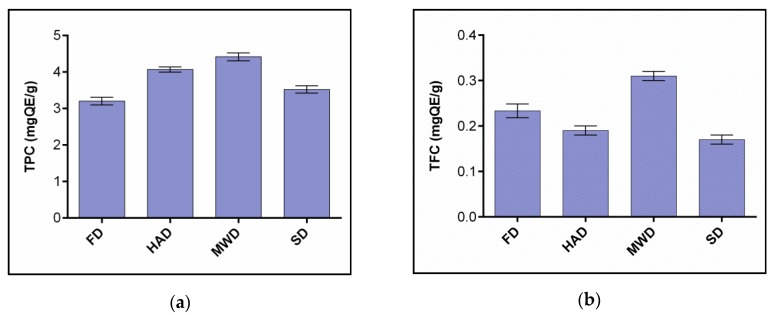
The content of total phenols (**a**) and total flavonoids (**b**) in the dry fruit bodies of *Pleurotus ostreatus* after in vitro digestion. FD—freeze drying; HAD—hot air drying; MWD—microwave drying; SD—sun drying.

The results showed that, in the gastrointestinal digestive model, MWD samples had the highest TPC values in vitro, followed by HAD, SD and FD samples. This is clearly shown in Figure 4.

The results obtained to some extent contradict the data from Lim, & Murtijaya [48], according to which intensive and rapid heating during microwave drying can lead to severe thermal degradation of phenolic compounds. It is also known that phenolic substances are found at the cellular level in vacuoles and are separated from oxidative enzymes in intact tissues [49]. Microwave drying is associated with the destruction of the cell structure of mushrooms and leads to the release of oxidative and hydrolytic enzymes and thus to the destruction of phenols. 

The ambiguity of the results shown by us can be quite logical. Many researchers [50] have noticed that the TPC content in different plant objects changes differently under various drying conditions. The drying process can lead to high or low TPC values depending on the type of plant material and the localization of the phenolic compounds present in the cell.

In our case, the maximum TPC value in MWD samples is probably determined both by high temperatures, which according to Dewanto et al. [51] can deactivate destructive enzymes, and by the best extraction of phenolic acids from mushroom tissue destroyed by microwave radiation. For example, Kubra and Rao [52] combine the increase of TPC in dried MWD plant products with microwave energy, leading to the degradation of cell components and the release of polyphenols from matrices. 

The loss of TPC in SD and FD mushroom samples can be caused by both enzymatic and non-enzymatic reactions of phenolic compounds during prolonged drying [53]. Low freeze-drying and solar drying temperatures are not capable of inactivating oxidative enzymes, which can lead to relatively low TPC values in FD and SD samples.

Samples of *Pleurotus ostreatus* dried fruit extracts by number of flavonoids (TFC) were distributed MWD-FD-HAD-SD in the following order.

Contrary to our assumption that low-oxygen and low-temperature FD media could effectively minimize flavonoid losses, the maximum TFC was recorded in MWD sample extracts. This is confirmed by the information provided by Toor and Savage [49] that the permeability of microwave radiation destroys the cellular components of tissue, making flavonoids more accessible for extraction. In addition, the relatively short heating time of microwave drying is more advantageous for the preservation of flavonoids than that of hot air drying. 

The lowest TFC values were recorded in SD samples. This is most likely related to the duration of the drying and thus to the oxidation processes, which has been repeatedly observed in the works of Lacramioara et al. [54] in their investigation of dehydrogenation processes in plants.

#### 3.3.3. Composition of Secondary Metabolites

Gas chromatography with mass spectroscopy was used to investigate the composition of secondary metabolites in dry oyster mushroom extracts. 

The chromatographic profile of the main substances identified, whose presence and content reflect the differences between the investigated samples of dry *Pleurotus ostreatus* mushroom extracts, is shown in Table 3.

Extracts from dry fruit bodies of *Pleurotus ostreatus* in simulated gastrointestinal digestion containing more than 50 compounds in the range of 0.22% to 48.62%, identified from the database of the NIST0,5a library. These include alcohols, aldehydes, acids and esters, heterocyclic compounds, fatty acids, amino acids, peptides, aromatic and other compounds. MWD samples had the largest number of identified substances. The relative percentage of many compounds was higher in MWD and FD extracts than in HAD and SD.

#### 3.3.4. MALDI-TOF Mass Spectrometry

**Figure 5:**
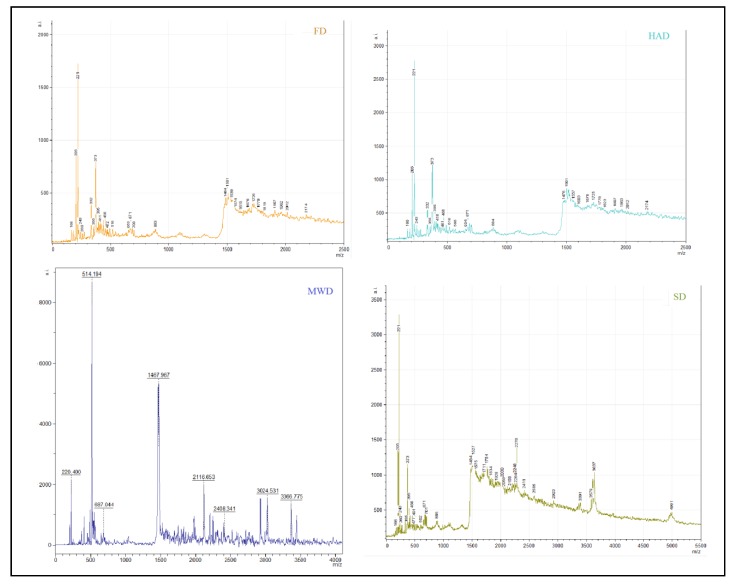
Data of the MALDI-TOF mass spectrometry of dry fruiting bodies *Pleurotus ostreatus* after in vitro digestion. FD—freeze drying; HAD—hot air drying; MWD—microwave drying; SD—sun drying.

The study of the peptide profile of the dry oyster mushroom after the imitation of digestion in vitro showed that the mass spectra of FD and HAD extracts included signals of varying intensity in the range of 160–2200 Da (Figure 5).

The range of MWD samples mass spectra was 180–3400 Da, SD samples 160–5000 Da. At the same time, 17 signals from m/z from 160 to 885 Da and 13 signals in the range of 1450–2200 Da were detected in FD and HAD samples. For MWD samples 12 signals were recorded in the range of 180–760 Da, 11 signals in the range of 1400–3400 Da. The scales of SD samples included 15 signals from m/z to 900 Da and about 20 signals in the range of 1400–5000 Da.

The peptide profile of dry fruit bodies of the oyster mushroom after digestion in the aspect of antioxidant and ACE inhibitory activities, obtained using the BIOPEP database, is presented in Table 4.

### 3.4. Influence of Drying Processes on the Antioxidative Activity of Pleurotus Ostreatus In Vitro for the Simulation of Gastrointestinal Digestion

There is not a single universal method capable of providing an accurate and comprehensive picture of the antioxidant profile, as several mechanisms underlying the antioxidant activity of mushrooms are identified, including the adjustment of the indirect chain reaction with free radicals, hydrogen donation, chelation of catalytic ions, removal of peroxides [36]. A single analysis is not sufficient to measure the antioxidant capacity, so a total of five methods were used in this study: ABTS radical scavenging activity, reducing performance activity, iron reducibility (the FRAP assay), total antioxidant capacity according to the electrochemical method, and lipid peroxidation inhibition test (Table 5).

ABTS analysis is often used to determine the total antioxidant capacity of individual compounds and complex mixtures of different plants [55]. According to the available tabular data, the average values of radical absorption activity obtained with the ABTS method for oyster mushroom extracts of dried FD, HAD, and SD did not differ statistically. The maximum radical-binding effect was observed in oyster mushroom extract dehydrated MWD and significantly exceeded the other samples in this indicator. The differences revealed despite the data from Duan and Xu [17], which indicate a lower safety of the antioxidant properties of mushroom dried MWD, can be explained by the destruction of cell walls by microwaves, which allow the availability of antioxidants for extraction. In addition, the relatively high antiradical activity of MWD samples may be associated with a high content of succinic acid (butanedioic acid, monomethyl ester), almost 14 times higher than that of FD, HAD and SD samples (see results of GC-MS above). This substance not only has an antioxidant effect alone, but also according to Kolupaev et al. [56] increases the activity of antioxidant enzymes: catalases and peroxidases.

The leading position of MWD extracts in the radical absorption activity of ABTS may also be due to the maximum number of peptides in their composition, consistent with the results of several researchers [35], which shows the relationship between antioxidant activity and mushrooms peptides. However, contrary to our assumption, the results of the MALDI-TOF mass spectrometric analysis of the peptide profile show a small representation of antioxidant peptides in MWD samples, which indicates other mechanisms of high activity of radical absorption of mushrooms obtained by microwave drying.

The reduction in performance activity of extracts in ascending order was distributed as follows: FD < SD < HAD < MWD. The results obtained of the largest manifestation of regenerative capacity of MWD samples are consistent with the data of Ji et al. [13] and their assumption that microwave-dried mushrooms have the ability to release electrons to reactive free radicals, transform them into more stable, non-reactive particles and terminate the chain reaction of free radicals.

The comparison of the ferric reduction capacity shows that the methods for drying FD and SD provide very accurate results (3.26 ± 0.08 mg/g; 3.38 ± 0.08 mg/g) with respect to the mushroom species studied. Of the four samples, MWD (5.03 ± 0.13 mg/g) and HAD (4.15 ± 0.10 mg/g) oyster mushroom extracts were the most effective in terms of iron restoration ability. The results obtained confirm the data of Ji et al. [13] and may be based on Dalmau et al. [18] and Heleno et al. [57] are caused by the enzymatic degradation of antioxidant compounds in the application of long-term dehydrogenation methods at low temperatures such as lyophilization and solar drying.

In addition, higher rates of iron reducing capacity of oyster mushroom extracts dried by MWD and HAD processes and characterized by heating may be associated with the release of phytochemical compounds caused by the thermal destruction of cell components and the formation of new substances with increased antioxidant potential, such as Maillard reaction products [58]. This is supported by literature reports on increased antioxidant activity after heat treatment of some raw materials such as tomatoes [51], mushrooms [59] and ginseng [60]. For example, according to Čechovská et al. [61], an increase in the antioxidant activity of plums after high temperature drying is achieved by the resulting substance 4H-pyran-4-on,2,3-dihydro-3,5-dihydroxy-6-methyl, which is the main product of the Maillard reaction, and in the extracts of the dry oyster mushrooms examined, only MWD samples were recorded (see above results of GC-MS).

The role of phenols in providing superior iron reduction capability of MWD and HAD samples, which are characterized by their highest content, is also possible. According to Ferreira et al. [47] and Muszyńska et al. [62] phenolic compounds can chelate elements (e.g., Fe, Cu) which can produce reactive oxygen species.

HAD extracts were the leading electrochemical method to assess the total antioxidant activity of dry oyster extracts. The remainder of the samples was distributed in the following order of acceptance FD > SD > MWD. Paradoxically, MWD extracts showing the highest antioxidant activity registered by spectrophotometric methods (Reducing Power Activity, ABTS, FRAP assay) were characterized by the lowest values of total antioxidant activity determined by the electrochemical method. This confirms that the already available data [63] may be associated with a low concentration of low molecular weight substances in the MWD extract, in particular, amino acids, whose antioxidant activity is the effect on electrochemical oxygen recovery effectively demonstrated by the above method. Thus, according to the above data, the largest amount of amino acids was found in HAD extracts (4.16 ± 0.18 g/L), the lowest in MWD samples (3.51 ± 0.15 g/L). According to the above data from GC-MS, only alanine and phenylalanine amino acids were found in HAD extracts and the concentrations of ornithine, leucine and uric amino acids were many times higher than in FD and SD samples. These amino acids were not identified at all in MWD extracts.

**Figure 6:**
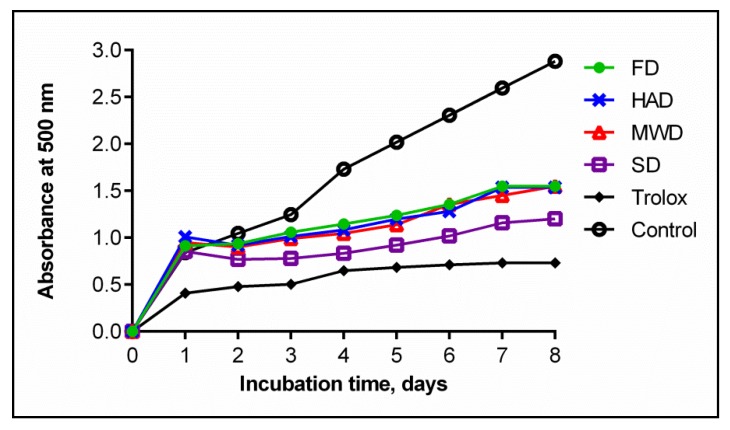
Lipid peroxidation inhibition of dry fruiting bodies *Pleurotus ostreatus* after in vitro digestion. Lower absorbance at 500 nm represents higher lipid peroxidation inhibition. FD—freeze drying; HAD —hot air drying; MWD—microwave drying; SD—sun drying.

No less interesting were the results of estimating the inhibition of lipid oxidation reactions. Samples of extracts from oyster mushroom dried SD were significantly differentiated by the degree of inhibition of lipid oxidation. The samples subjected to other drying methods had no statistically significant differences in the value of this activity (Figure 6).

Phenolic substances in mushrooms are known to play an important role in inhibiting lipid oxidation [64]. In our study, there was no correlation between phenol concentration and activity to block lipid oxidation processes.

This may indicate the role of other secondary metabolites in inhibiting lipid oxidation reactions, the content of which, according to Kim et al. [65], increases the process of drying mushrooms in the sun by activating specific enzymes that catalyze the biosynthesis of secondary metabolites by ultraviolet radiation. According to the above results of GC-MS extracts such substances may be: pyrrolo[1,2-a]pyrazine-1,4-dione,hexahydro-3-(2-methylpropyl)-; dl-valine; 2-dimethylsilyloxytetradecane; d-mannitol,1,4-anhydro-; dl-proline,5-oxo-; 4-cyanobenzoic acid, 2-phenyl ethyl ester. These compounds were much less numerous in FD, HAD and MWD samples than in SD-dried mushrooms or were not present at all. This is partially confirmed by Manimaran & Kannabiran [66], which indicates the ability of some of these substances to participate in antioxidant reactions.

### 3.5. ACE Inhibitory Activity

ACE inhibitory activity of mushrooms, the genus *Pleurotus*, is confirmed by a number of studies. Available data on ACE inhibitory activity focus on the action of proteins and bioactive peptides [67,68].

**Figure 7:**
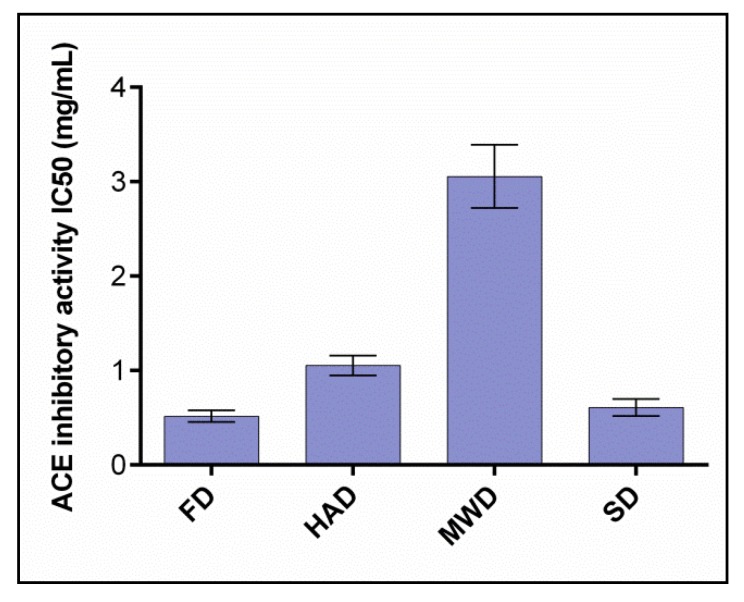
ACE inhibitory activity IC50 (mg/mL) of dry fruit bodies *Pleurotus ostreatus* after in vitro digestion. FD—freeze drying; HAD—hot air drying; MWD—microwave drying; SD—sun drying.

ACE inhibitory activity of oyster mushroom dried in various ways was evaluated after the simulation of gastrointestinal digestion. The results were expressed in IC50 units showing the concentration of the substance required to inhibit 50% of ACE activity (Figure 7).

The obtained values were correlated with the results of other researchers [36] and once again demonstrated the potential of oysters for ACE inhibition. At the same time, it was found that the activity of ACE-inhibiting mushrooms after the simulation of gastrointestinal digestion differed significantly depending on the drying method used. FD mushrooms showed maximum activity with respect to ACE IC50 inhibition (0.51 ± 0.03 mg/mL). The value of ACE IC50 oyster mushroom inhibition activity dried HAD and SD was 1.07 ± 0.06 mg/mL and 0.61 ± 0.05 mg/mL, respectively. The lowest ACE inhibitory activity was shown by dried MWD mushrooms (3.04 ± 0.19 mg/mL). Probably, heating and microwave radiation lead to the destruction of active proteins and peptides of mushrooms with the property of ACE inhibition. This is logically confirmed by the lowest ACE representation of inhibitory peptides in MWD samples described above and echoes the results of the proteomic analysis performed by Lau [69], according to which lyophilic drying provides high ACE inhibitory activity of mushroom sublimates due to the preservation of a wide range of proteins, including low-molecular weight proteins, which are potential ACE inhibitors.

In addition, the minimal ACE inhibition activity of dried MWD mushrooms can also be explained by the high-temperature inactivation of the proteinase inhibitors contained in the mushrooms, which block the activity of gastrointestinal enzymes and, as a result, increased the digestive damage of ACE-active protein and peptide inhibition.

## 4. Conclusions

In this study it was found that various methods for drying fruiting bodies of *Pleurotus ostreatus* have different effects on the structural properties, rehydration capacity and thus on the chemical composition and antioxidant profile of the dry product in experimental gastrointestinal digestion. *Pleurotus ostreatus* studied showed good antioxidant capacity and potential antihypertensive effect as demonstrated by their inhibitory effect towards ACE.

Using different methods of estimating antioxidant activity based on different chemical reactions, the peculiarities of the antioxidant properties of *Pleurotus ostreatus* fruiting bodies dried in various ways were identified and the most effective methods of drying to obtain oyster mushrooms with the greatest antioxidant effect were determined for different application points of antiradical function. It was found that under the conditions of the gastrointestinal digestive model in vitro the dry oyster mushroom as a food product or its component has the highest radical absorption activity after microwave drying. The reduction of performance activity and iron-reducing ability of oyster mushroom is maximally preserved in microwave drying and hot-air drying. Antioxidant properties based on the mechanisms of electrochemical oxygen recovery manifest most in mushrooms dewatered with hot air. The properties of the antioxidant with emphasis on a high activity of inhibiting lipid oxidation of the oyster mushroom are largely preserved after drying in the sun. After the simulation of gastrointestinal digestion, mushrooms dried lyophilically and in the sun showed relatively high ACE inhibitory activity.

At the same time, we cannot ignore the fact that the drying methods used in this study differ significantly in terms of process complexity, energy consumption and, consequently, cost. The choice between drying methods will therefore depend on the final product. For example, if the intention is to obtain a dry oyster mushroom product with high ABTS radical scavenging activity, reducing power activity and ferric reducing ability, then microwave drying may be the relatively best choice. Drying in a microwave oven compared to hot air drying significantly reduces the drying time and is more energy efficient. If, however, there is a need for a product with a pronounced electrochemical oxygen recovery capability and a relatively high ACE-inhibiting activity, hot air drying, despite its higher cost, is the best option.

In the case of a dry oyster mushroom product with an emphasis only on the maximum ACE-inhibiting activity, of the two methods of drying: lyophilic and solar drying, it would be more rational to choose the latter. The method of drying in the sun compared to lyophilic drying is simple, has no special requirements for expensive equipment and is characterized by significantly lower costs.

The complex and costly method of lyophilic drying of *Pleurotus ostreatus* is not excluded and can be considered as an alternative in the case of obtaining a substance with high ACE-inhibiting activity in the production of complex biopreparation, in order to preserve thermolability and to stabilize the action of oxygen and UV irradiation substances that provide other properties of oyster mushrooms, not considered in this study.

Thus, the experimental substantiation of separate drying methods provides an antioxidant or ACE inhibitory effect when obtaining a dry product of oyster mushrooms as a potential functional food product or an ingredient with the maximum expression of the whole complex or separate properties.

## Figures and Tables

**Table 1:** Microtomographic parameters of the fruit bodies of *Pleurotus ostreatus*, dried in various ways.

	POV ^1^	OSVR ^2^	FI ^3^	DA ^4^	SMI ^5^
**FD**	87.14 ± 3.78 ^a^	0.015 ± 0.00067 ^a^	−0.08 ± 0.0034 ^a^	0.26 ± 0.010 ^a^	−32.08 ± 1.39 ^a^
**HAD**	72.34 ± 3.20 ^b^	0.019 ± 0.00081 ^b^	0.000029 ± 0.0000012 ^b^	0.37 ± 0.016 ^b^	0.009 ± 0.00034 ^b^
**MWD**	56.76 ± 2.53 ^c^	0.018 ± 0.00077 ^b^	0.00082 ± 0.00034 ^c^	0.55 ± 0.024 ^a^	0.27 ± 0.012 ^c^
**SD**	59.93 ± 2.58 ^c^	0.021 ± 0.0011 ^b^	−0.014 ± 0.00060 ^d^	0.34 ± 0.014 ^b^	−0.38 ± 0.016 ^d^

Different superscript letters indicate statistically significant differences between the means (*p* < 0.05) for each parameter. ^1^ Percent object volume; ^2^ Object surface/volume ratio; ^3^ Fragmentation index; ^4^ Degree of anisotropy; ^5^ Structure modeling index. FD—freeze drying; HAD—hot air drying; MWD—microwave drying; SD—sun drying.

**Table 2:** Some physicochemical parameters of the dry fruiting bodies of *Pleurotus ostreatus* after in vitro digestion.

The Investigated Parameter	FD	HAD	MWD	SD
Amount of dry matter, g/L	40.0 ± 1.73 ^a,b^	37.3 ± 1.61 ^a^	43.3 ± 1.92 ^b^	39.9 ± 1.82 ^a,b^
Ionometry (pH)	7.05 ± 0.31	6.91 ± 0.29	7.5 ± 0.33	6.93 ± 0.31
Total nitrogen (TN),%	0.27 ± 0.01 ^a^	0.20 ± 0.01 ^a^	0.32 ± 0.01 ^b^	0.22 ± 0.01 ^a^
Amine nitrogen (AN), g/L	0.87 ± 0.04 ^a^	0.95 ± 0.04 ^a,c^	0.80 ± 0.04 ^b^	1.01 ± 0.04 ^c^
Degree of hydrolysis (DH),%	32.1 ± 1.56 ^a^	46.7 ± 2.08 ^b^	25.1 ± 1.21 ^c^	45.8 ± 2.08 ^b^
Amount of amino acids, g/L	3.81 ± 0.17 ^a^	4.16 ± 0.18 ^b^	3.51 ± 0.15 ^a^	4.02 ± 0.19 ^b^
Amount of peptides, g/L	8.06 ± 0.35 ^a^	4.73 ± 0.21 ^b^	10.46 ± 0.45 ^c^	5.23 ± 0.21 ^b^
Amount of monosaccharides in recalculation for glucose,%	0.24 ± 0.01 ^a^	0.21 ± 0.01 ^a^	0.23 ± 0.01 ^a^	0.22 ± 0.01 ^a^

Different superscript letters indicate statistically significant differences between the means (*P* < 0.05) for each parameter. FD-freeze drying; HAD-hot air drying; MWD-microwave drying; SD-sun drying.

**Table 3:** Profile of secondary metabolites of dry fruiting bodies *Pleurotus ostreatus* (based on the results of gas chromatography-mass-spectroscopy) after in vitro digestion.

Name of the Compounds	Structure	Relative Peak Area (%)			
		FD	HAD	MWD	FD
l-Lactic acid	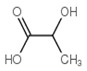	48.72	22.24	27.23	28.81
Butanedioic acid, 2,3-dihydroxy- [R-(R*,R*)]-, dimethyl ester	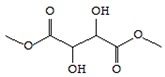	nd	0.17	1.79	nd
Glycerin	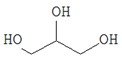	6.10	2.08	3.15	2.08
6-Nitrohexan-2-ol	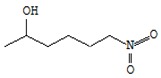	nd	1.14	nd	nd
1-Butanamine, 3-methyl-*N*-(3-methylbutylidene)-	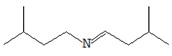	nd	nd	1,23	nd
Cyclopentane, 1-acetyl-1,2-epoxy-	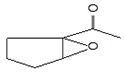	nd	nd	1,11	nd
1-Propanol, 2-(2- hydroxypropoxy)-	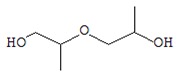	2.19	1.65	2.88	1.61
2-Propanol, 1,1’-oxybis-	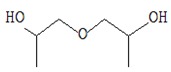	1.19	1.11	2.2	0.96
Propanamide, *N*-methyl-2-amino-	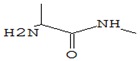	nd	nd	nd	1.49
Benzeneethanamine	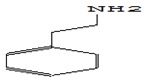	2.14	1.72	nd	1.92
Butanedioic acid, monomethyl ester	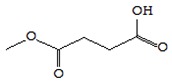	0.98	0.61	14.78	0.87
2(3H)-Furanone, dihydro-4-hydroxy-	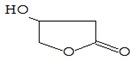	nd	nd	1.24	nd
Ornithine	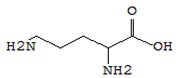	3.09	6.74	nd	2.85
Benzeneacetic acid	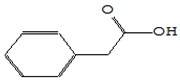	nd	4.56	nd	0.45
dl-Valine	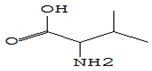	nd	nd	nd	1.99
Alanine	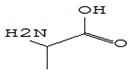	nd	5.31	nd	nd
1,3-Dimethyl-3,4,5,6-tetrahydro-2(1H)-pyrimidinone	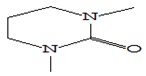	1.38	nd	nd	nd
d-Leucine	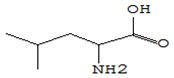	0.65	7.26	nd	2.54
2-Dimethyl silyloxytetradecane	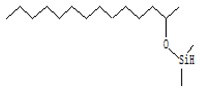	nd	nd	nd	5.3
d-Mannitol,1,4-anhydro-	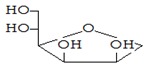	nd	nd	nd	7.52
Uracil	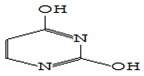	0.37	4.12	nd	nd
1,3-Dimethyl-3,4,5,6-tetrahydro-2(1H)-pyrimidinone	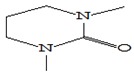	1.58	nd	nd	1.67
4H-Pyran-4-one, 2,3-dihydro-3,5-dihydroxy-6-methyl-	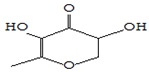	nd	nd	2.89	nd
1-Acetyl-4-piperidinecarboxylic acid	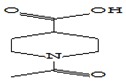	nd	2.0	nd	nd
dl-Phenylalanine	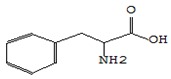	nd	3.3	nd	nd
dl-Proline,5-oxo-	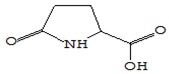	4.01	2.68	1.64	4.28
Adenosine 3’,5’-cyclic monophosphate	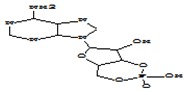	nd	nd	2.71	2.67
4-Cyanobenzoic acid, 2-phenylethyl ester	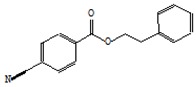	nd	nd	nd	1.19
*N*-(gamma-l-Glutamyl) phenylalanine	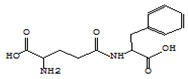	3.38	nd	nd	nd
3-Methyl-1,4-diazabicyclo [4.3.0] nonan-2,5-dione, *N*-acetyl-	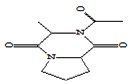	1.28	nd	nd	0.94
dl-Alanyl-l-leucine	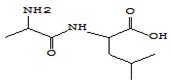	1.03	3.74	nd	1.71
Pyrrolo [1,2-a] pyrazine-1,4-dione, hexahydro-3-(2-methylpropyl)-	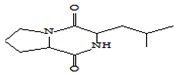	3.19	2.68	2.59	5.49
5,10-Diethoxy-2,3,7,8-tetrahydro-1H,6H-dipyrrolo[1,2-a:1’,2’-d] pyrazine	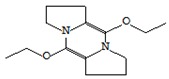	2.29	1.89	1.65	1.0
2,4-Imidazolidinedione, 5-(4-hydroxybutyl)-	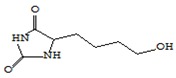	nd	0.53	nd	1.11
n-Hexadecanoic acid	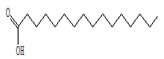	2.43	1.17	2.50	1.24
9,12-Hexadecadienoic acid, methyl ester	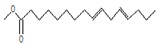	nd	nd	nd	1.15
9,12-Octadecadienoic acid (Z,Z)-	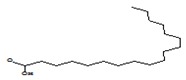	3.53	nd	3.97	nd
Hexadecanoic acid, 2-hydroxy-1-(hydroxymethyl)ethyl ester	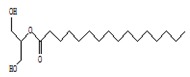	nd	nd	2.99	nd
9,12-Octadecadienoic acid (Z,Z)-, 2,3-dihydroxypropyl ester	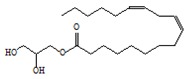	1.42	nd	2.96	nd
Ergosterol	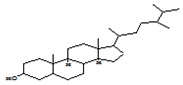	1.68	nd	nd	0.56
Pyrrolo[1,2-a] pyrazine-1,4-dione, hexahydro-3-(phenylmethyl)-	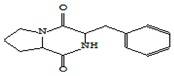	nd	1.39	0.51	0.58

nd: not detected. Identified substances with a relative peak area less than 0.5% were not listed in the table. FD—freeze drying; HAD—hot air drying; MWD—microwave drying; SD—sun drying.

**Table 4:** Characterization of the peptide profile of the dry fruit bodies of *Pleurotus ostreatus* after in vitro digestion (in accordance with the BIOPEP database).

Chemical Mass, Da	ID	Sequence	Activity	FD	HAD	MWD	SD
243	3342	GPA	ACE inhibitor	+	+	−	+
	7810	KP	ACE inhibitor	+	+	−	+
	7837	PQ	ACE inhibitor	+	+	−	+
	9041	AGP	ACE inhibitor	+	+	−	+
	8218	KP	antioxidative	+	+	−	+
355	8000	LHS	antioxidative	+	+	−	+
373	7654	NKL	ACE inhibitor	+	+	+	+
395	8220	TFE	antioxidative	+	+	−	+
405	3301	HLH	antioxidative	−	−	+	−
	3302	LHH	antioxidative	−	−	+	−
	7909	IHH	antioxidative		−	+	−
	7919	NHH	antioxidative	−	−	+	−
	7984	HIH	antioxidative	−	−	+	−
	8017	LWS	antioxidative	−	−	+	−
	8225	SWN	antioxidative	−	−	+	−
	9179	QYP	antioxidative	−	−	+	−
	9190	MAW	ACE inhibitor	−	−	+	−
	9350	TTW	ACE inhibitor	−	−	+	−
456	7652	KFY	ACE inhibitor	+	+	−	+
472	3364	HGLF	ACE inhibitor	+	−	−	+
	7551	YQY	ACE inhibitor	+	−	−	+
	7651	YKY	ACE inhibitor	+	−	−	+
	7653	KYY	ACE inhibitor	+	−		+
	7931	KYY	antioxidative	+	−	−	+
	7934	YKY	antioxidative	+	−	−	+
	7937	YYK	antioxidative	+	−	−	+
	7948	YYQ	antioxidative	+	−	−	+
	7966	QYY	antioxidative	+	−	−	+
	7969	YQY	antioxidative	+	−	−	+
477	3364	HGLF	ACE inhibitor	+	−	−	+
	7551	YQY	ACE inhibitor	+	−	−	+
	7651	YKY	ACE inhibitor	+	−	−	+
491	7904	SALAM	antioxidative	−	+	−	+
	7943	YYF	antioxidative	−	+	−	+
	7961	FYY	antioxidative	−	+	−	+
	7963	YFY	antioxidative	−	+	−	+
	8431	MGSPT	antioxidative	−	+	−	+
	9070	MRW	ACE inhibitor	−	+	−	+
516	8078	RWR	antioxidative	+	+	+	−
552	9242	PLPLL	ACE inhibitor	−	−	+	−
566	7485	TKVIP	ACE inhibitor		+	−	−
602	8278	VPYPQ	antioxidative	−	−	−	+
	8963	VPVST	antioxidative	−	−	−	+
654	3566	EPKAIP	ACE inhibitor	+	+	−	−
	3970	WLAHK	ACE inhibitor	+	+	−	−
	9217	TFPHGP	ACE inhibitor	+	+	−	−
	8430	HVAGTVA	antioxidative	+	+	−	−
671	3367	GKKVLQ	ACE inhibitor	+	+	−	+
	9099	MTEEY	ACE inhibitor	+	+	−	+
	9109	LIWKL	ACE inhibitor	+	+	−	+
	9100	MTEEY	antioxidative	+	+	−	+
687	2651	VLPYPV	ACE inhibitor	−	−	−	+
	2667	LHLPLP	ACE inhibitor	−	−	−	+
	3575	QPQAFP	ACE inhibitor	−	−	−	+
	7568	KVREGT	ACE inhibitor	−	−	−	+
	8730	RWAEK	antioxidative	−	−	−	+
699	3420	GVYPHK	ACE inhibitor	−	−	+	−
	8305	QLGNLGV	antioxidative	−	−	+	−
	8950	WCTSVS	antioxidative	−	−	+	−
701	9443	AGDDAPR	antioxidative	+	−	−	+
	9444	GKDAVIV	antioxidative	+	−	−	+
	8306	RDVPSLM	antioxidative	+	−	−	+
	9449	IDDVLK	antioxidative	+	−	−	+
884	9224	MPVHTDAD	ACE inhibitor	+	+	−	+
	9445	AIGVGAIEP	antioxidative	+	+	−	+
	9531	SNLCRPCD	antioxidative	+	+	−	+
	8448	WHNVSGSP	antioxidative	+	+	−	+
1615	8100	LKQELEDLLEKQE	antioxidative	+	−	−	−
1726	3809	LQSGDALRVPSGTTYY	antioxidative	+	+	−	+
1754	9240	LVYPFPGPIPNSLPQN	ACE inhibitor	−	−	−	+
	9370	VKRRGQDCIHGFCSD	antioxidative	−	−	−	+
1778	8464	LVMFLDNQHRVIRH	antioxidative	−	+	−	−
2012	9201	DPAQPNYPWTAVLVFRH	antioxidative	+	+	−	−

FD—freeze drying; HAD—hot air drying; MWD—microwave drying; SD—sun drying.

**Table 5:** ABTS radical scavenging activity, reducing power activity and ferric reducing ability (the FRAP assay) of dry fruit bodies *Pleurotus ostreatus* after in vitro digestion (M ± m).

Drying Method	ABTS Radical Scavenging Activity μmolTrolox/g	Reducing Power Activity mg Equivalent to Ascorbic Acid/g	Ferric Reducing Ability mg Equivalent to Ascorbic Acid/g	Total Antioxidant Capacity by the Electrochemical Method mg Equivalent to Gallic Acid/L
**FD**	24.0 ± 1.04 ^a^	8.76±0.36 ^a^	3.26 ± 0.14 ^a^	9.04 ± 0.26 ^a^
**HAD**	24.8 ± 1.23 ^a^	12.4 ± 0.54 ^b^	4.15 ± 0.17 ^b^	10.99 ± 0.21 ^b^
**MWD**	28.6 ± 1.25 ^b^	14.2 ± 0.62 ^c^	5.03 ± 0.23 ^c^	7.09 ± 0.38 ^c^
**SD**	25.4 ± 1.10 ^a^	9.8 ± 0.43 ^d^	3.38 ± 0.14 ^a^	7.39 ± 0.28 ^c^

Different superscript letters indicate statistically significant differences between the means (*p* < 0.05) for each parameter. FD—freeze drying; HAD—hot air drying; MWD—microwave drying; SD—sun drying.
